# Dexmedetomidine leads to the amelioration of stress response to surgery

**DOI:** 10.12669/pjms.40.9.8792

**Published:** 2024-10

**Authors:** Khalida Ajmal, Akbar Waheed, Rashada Farooqi, Qaisar Mansoor

**Affiliations:** 1 Dr. Khalida Ajmal, MBBS, M.Phil., CHPE, Department of Pharmacology, Wah Medical College, Wah Cantt, Pakistan; 2 Dr. Akbar Waheed, MBBS, MPhil, Ph.D., CHPE. Dept. of Pharmacology, Islamic International Medical College, Riphah University, Rawalpindi, Pakistan; 3 Dr. Rashada Farooqi, Department of Anesthesia, POF Hospital, Quaid Avenue Wah Cantt, Rawalpindi, Pakistan; 4 Dr. Qaisar Mansoor, Institute of Biomedical & Genetic Engineering, Islamabad, Pakistan

**Keywords:** Dexmedetomidine (Dex), Tumor Necrosis Factor alpha (TNF-α), Interleukin-6 (IL-6), Cortisol (Cor), Ear, nose and throat (ENT)

## Abstract

**Objectives:**

To determine the impact of intravenous Dexmedetomidine (Dex) administered perioperatively on stress response markers in patients undergoing ENT surgeries.

**Methods:**

This randomized interventional study was conducted at POFs Hospital and NUMS affiliated WMC, Wah Cantt, in collaboration with IIMC Rawalpindi, Pakistan, from October 2021 to April 2022. One hundred patients aged between 15-60 years, after satisfying the inclusion standards were randomly divided into two groups C and D. Group-C (n=50) received normal saline in addition to the standard anesthesia protocol. The intervention Group-D (n=50) was administered 1µg/kg dexmedetomidine hydrochloride intravenously over 10 minutes just before the induction followed 0.5μg/kg/hr as maintenance dose till the end of surgery. Serum inflammatory biomarkers (interleukins-6, TNF-α and cortisol) were measured in blood samples in both groups, taken at 0 (T0), 30 minutes(T1), and two hours (T2) time intervals and analyzed by using Enzyme-linked immunosorbent assay (ELISA). Data was statistically analyzed using SPSS 23.

**Results:**

The patients receiving Dex showed marked decrease in serum levels of cortisol, TNF- α and interleukins-6, from 139.73 to10.18, 99.51 to 0.96 and 85.09 to 0.96 respectively. Comparison between C and D groups revealed significant differences (p≤0.05) in these serum biomarkers.

**Conclusions:**

In the present study, intravenous Dex suppressed the intraoperative rise in levels of cytokine secretion and enhanced smooth recovery with no incidence of nausea and vomiting. These effects could be attributed to the anti-inflammatory effects of dexmedetomidine.

***Iranian Registry of Clinical Trials reference:*** IRCT20230824059251N1.

***Abbreviation:***

***ENT:*** Ear, Nose and Throat, ***BMI*:** Body Mass Index, ***MAC:*** Monitored Cared Anesthesia, ***ASA*:** American Society of Anesthesiologists, ***SPSS:*** Statistical Package for Social Sciences, ***SD*:** Standard Deviation, ***PONV:*** Postoperative Nausea and Vomiting, ***OT*:** Operation Theater, ***POFs:*** Pakistan Ordinance Factories, ***WMC:*** Wah Medical College, ***IIMC:*** Islamic International Medical College, ***NUMS:*** National University of Medical Sciences.

## INTRODUCTION

Surgical procedures are considered potential triggers for the secretion of pro-inflammatory cytokines and cortisol owing to activation of the sympathetic nervous system during and immediately after surgery. Cytokines production is related to the degree of surgical tissue injury and have major role in immunity and inflammation. Undue increase in these cytokines (IL-6, TNF- α) and cortisol release may cause fever and other organ dysfunction later after surgery. ENT surgeries especially tonsillectomies, are coupled with dysfunctional immune response. Preoperative administration of dexmedetomidine intravenously (IV) inhibits the inflammatory responses.^1^

Dexmedetomidine, a congener of clonidine, is centrally acting and highly selective agonist at α_2A_ adrenoceptor (ADRA2A). It is being increasingly used as an important modulator of anxiety and pain. It decreases perioperative stress and inflammation but preserves immune function in surgical patients. Dexmedetomidine is opiate-sparing, sedative-analgesic with sympatholytic properties. It is more frequently used by anesthetists to prevent common problems in the peri-operative period. Central sympatholytic effects of dexmedetomidine alleviate cytokines secretion secondary to stress induced sympathetic activation and immune system interactions.^2^ During surgery it is proved to exhibit hemodynamic stability, decreased rescue analgesia and lower required dose of other anesthetics, with no post-operative nausea, vomiting or respiratory depression and emergence phenomena. It provides conscious sedation and has been used as a sole agent to provide Monitored Anesthesia Care (MAC) in various surgical interventions.^3^ It’s anti-sialagogue activity facilitates easy intubation, blunt hemodynamic pressor response to laryngoscopy and intubation.^4^ Its beneficial effects range from pediatrics population to elderly patients.^5^,^6^ More recently dexmedetomidine is being used at the end-of-life intractable cancer pain management for pediatric and adult patients decreasing the requirement for rescue analgesia and improved the quality of communication between patients and their families.^7^

Dexmedetomidine has been used in our population in most of the neurological and other procedures and in spinal anesthesia.^8^ It proved to produce bloodless field under MAC in septoplasty^9^ and sinus endoscopic surgery,^10^ myringoplasty and tympanoplasty^11^ in India. However, its cytoprotective and anti-inflammatory properties are not studied in our region although it is documented to produce immunomodulatory effects associated with reduced cytokine production when used in critical ill Covid-19 patients and in cardiac surgeries.^12^

We initially conducted a successful pilot study to use Dex as sole anesthetic agents. It is linked with higher patient and surgeon satisfaction providing bloodless field during ENT surgeries. The favorable outcomes of this study can have appreciable economic impact on our low health budget population.

Currently limited data is available on anti-inflammatory and immunomodulatory aspects of Dex. None of the studies have estimated Cor, IL-6 and TNF-α concentration in serum possibly responsible for decreasing surgical stress response in Pakistan. We aimed to estimate these biomarkers quantitatively in serum of patients undergoing these surgeries. To avoid the effects of circadian rhythm of cortisol, all surgeries were planned to start after 1000 hours in the morning.

## METHODS

This prospective, interventional study was conducted at WMC affiliated tertiary care POFs Hospital, Wah Cantt, in collaboration with IIMC Islamabad, Pakistan, from October 2021 to April 2022. To ensure study power of 80% and to detect a difference of ≥20% in cumulative numbers of inflammatory markers, a sample size of 100 was calculated.^13^

### Inclusion & Exclusion Criteria:

The inclusion criteria of American Society of Anesthesiologists (ASA) physical status I and II, was adopted for undergoing tonsillectomy with general anesthesia. Those who refused to volunteer and with co-morbidities were excluded.

### Ethical Approval:

We got approval from IRB/IRC (Appl# Riphah/IRC/20/101).

**Fig.1:**
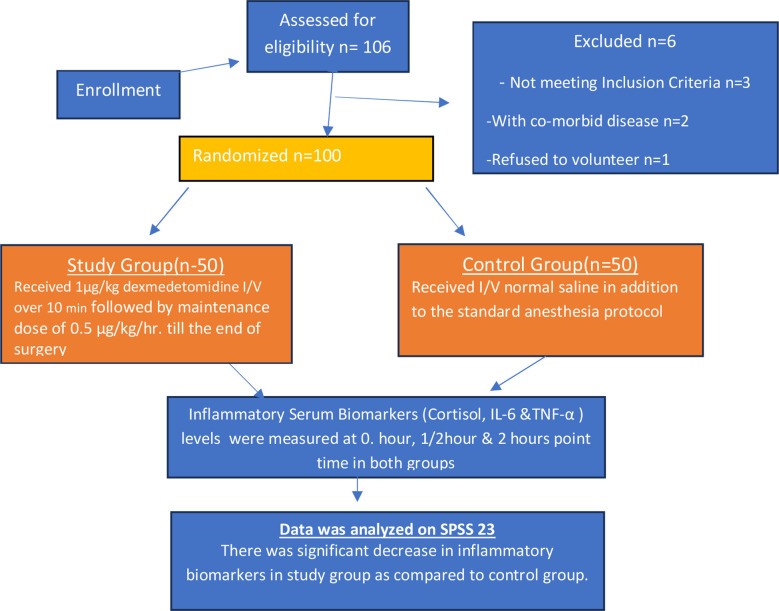
Schematic Consort e chart for study.

After getting written informed consent, the patients aged between 10-60 years were randomized into control Group-C (n=50) and intervention or drug Group-D (n=50). The Group-D was administered 1µg/kg dexmedetomidine hydrochloride intravenously over 10 minutes diluted in 20 ml of N/saline before the induction, followed by 0.5 μg/kg/hr as maintenance dose till the end of surgery. Group-C received normal saline in addition to the standard anesthesia protocol (propofol 2mg/kg and atracurium 0.6mg/kg, atropine and nalbuphine, Fig.1.^14^

Serum inflammatory biomarkers (Cor, TNF-α and IL-6) were measured in blood samples taken at 0 (To), 30 minutes (T1), and two hours (T2) intervals and assayed using commercially available enzyme-linked immunosorbent assay (ELISA) kits (R&D Systems, USA) according to the manufacturer’s instructions. Data was statistically analyzed using SPSS 23. Data was further computed applying ANOVA (one-way analysis of variance) and post-hoc Bonferroni test to calculate Means, SEM and standard deviation. *P*-value at or less than <0.05 was considered statistically significant.

## RESULTS

Among all the 100 patients in both groups, no statistically significant difference was found as regarding age, sex, and BMI (Table-I).

**Table-I:** Demographic and General Characteristics of the patients included in the study (N=100).

Characteristics of Patients	Control Group(C)n=50 Means ± SEM	Dex Group(D)n=50 Means ± SEM	P value
Age of Patients	25.9±1.6	26.8 ± 1.7.3	<0.686
BMI of Patients	22.5±0.38	23.5±0.45	<0.095
ASA Grading	2.00±0.00	1.00±0.00	<0.003
Duration of Surgery (minutes)	43.5±1.1	44.8±1.1	<0.005
Duration of Anaesthesia (minutes)	53.5±0.62	52.4±0.52	<0.001*
***Gender***	n=50	n=50	0.163
Females	35	41	76
Males	15	9	24
***Provinces***			**Total**
Sindh	4	6	10
Punjab	30	26	56
Baluchistan	6	6	12
KPK	10	12	22

### Inflammatory biomarkers:

(Plasma Cortisol ng/ml, Interleukin-6 pg/ml, and TNF-α pg/ml)

**Fig.2:**
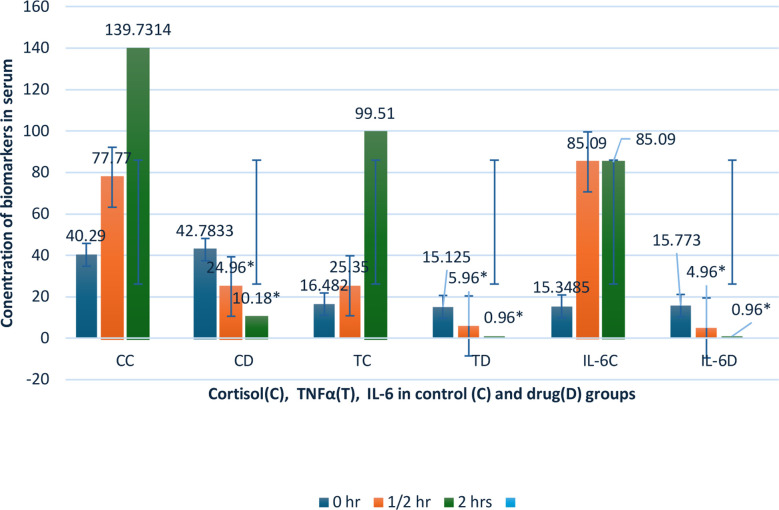
Graphic depiction of biomarkers with the standard deviation in both groups.

The baseline levels (T0) of Plasma Cortisol ng/ml, Interleukin-6 pg/ml, and TNF-α pg/ml in the two groups were not significantly different with the mean value in control group and in Dex group from 40.29 to 42.7833, 16.482 to 15.125 and 15.35 to 15.77 respectively (value of P>0.05). While concentrations at 30 minutes (T1) timepoint were significantly lower than T0 (P< 0.05) (Table-II). There was a significant lowering of cortisol, TNF-α and IL-6 in Dex group at two hours (T2) samples as compared to control group (from 139.73 to10.18, 99.51 to 0.96 and 85.09 to 0.96 respectively with value of P <000. Collectively stating we observed highly significant difference in the both groups as regard time of sampling except at baseline or (To) time (Table-II and Fig.2).

**Table-II:** Differences in serum levels of Cortisol, IL-6, TNF-α across the study.

Biomarkers at different timepoints	Control Group(C) n=50 Means ± SEM	Dex Group (D) n=50 Means ± SEM	P value
Cortisol (ng/ml) levels at 0 hrs.	40.2 ± 9.8	42.7 ±6.5	<0.56
Cortisol (ng/ml) levels at 30 mins	77.70±0.55	24.9±.01	<0.000*
Cortisol (ng/ml) levels at 2 hrs.	139.73±0.83	10.18±.04	<0.000*
TNF-α (pg/ml) levels at 0 hrs.	16.48±3.9	15.12±0.125	<0.92
TNF-α (pg/ml) levels at 30 mins	25.35±0.14	5.9±0.19	<0.000*
TNF-α (pg/ml) levels at 2 hrs.	99.5±1.03	0.96±0.019	<0.000*
IL-6 (pg/ml) levels at 0 hrs.	15.34±1.15	15.77±0.46	<0.99
IL-6 (pg/ml) levels at 30 mins	85.10±1.38	4.96±0.19	<0.000*
IL-6 (pg/ml) levels at 2 hrs.	85.09±1.38	0.96±0.12	<0.000*

### Rescue analgesia and smooth recovery:

Only 10% of patients in the study group called for analgesic and no atropine was given to patients of study group.

### Hemodynamic stability:

Objectively required degree of small decrease in blood pressure and heart rate was observed in study group throughout the intraoperative period but there were significant fluctuations in control group.

## DISCUSSION

The present study was conducted to document anti-inflammatory effects of Dexmedetomidine (Dex) by quantifying the serum levels of Cortisol, TNF-α and IL-6 in Pakistani patients undergoing ENT surgeries (Tonsillectomies) from all provinces with no discrimination of gender.

In our study, IV administration of dexmedetomidine pre-operatively resulted in significantly decreased levels of serum stress biomarkers. The baseline levels (T0) of Cortisol, IL-6 and TNF-α in Control group and in Dex group were almost same and not significantly different (P value > 0.05) while concentrations of these biomarkers at 30 minutes (T1) and two hrs. (T2) timepoints were significantly lower than T0 (P< 0.05) showing the definite efficacy of Dex as anti-inflammatory agents.

The physical and psychological trauma of surgery and anesthesia rapidly induces pro-inflammatory cytokines through sympathetic nervous system stimulation and releases TNF-α and IL- 6 into circulation which peaks at 30 minutes post-surgery. These markers may continue increasing and attain the maximum level in 1-3 hrs. and stayed for a few days without any intervention. TNF-α and IL-6 are key cytokines involved in cascade of acute phase response generation. TNF-α can cross blood brain barrier and may cause fever and cognitive dysfunction. The extent and severity of inflammation and tissue injury is proportionate to increase in IL-6 and TNF-α levels.^15^-^17^

Dexmedetomidine (DEX) is a highly specific agonist at α_2A_ receptors, leads to central sympatholytic effect by acting on α_2A_ receptors in lateral reticular nucleus. Decreased sympathetic outflow blocks the link for release of cortisol and cytokines. It produces hypnosis via α_2A_ receptors in locus coeruleus and analgesic effects from receptors present in substantia gelatinosa of dorsal horn of spinal cord involved in release of endogenous opioids. Dex paradoxically stimulates antimuscarinic effects via vagal stimulation^18^. Various studies in Pakistan have been carried out on use of Dexmedetomidine as an effective and safe anesthetic adjuvant and sedative but none of them has explored its ant-inflammatory effects.^7^-^10^,^19^

In the current study, I/V dexmedetomidine given preoperatively resulted in a more marked decrease of Cortisol, TNF-α and IL-6 levels in samples taken at two hours. interval as compared to control group with statistically significant value of P < 0.000. The same results were demonstrated in another study^20^ where dexmedetomidine was given continuously during open heart surgery and it suppressed intra-operative and post-operative cytokine surge, also amended post-operative inflammatory response profiles and sympathetic hyperalgesia.

Our findings are also consistent with an Australian study,^17^ they documented that preoperative administration of Dex in patients of delirium suppressed the TNF production and the release of mediators causing inflammation or sepsis.

The finding of randomized control studies carried out by Chinese authors^21^, established that dexmedetomidine alone or in combination with lidocaine used pre-operatively, alleviated inflammatory responses depicted in decreased level of IL-6, IL-1 and TNF-α during laparoscopic hysterectomy. Another study conducted by Chinese researchers^22^ appreciated valuable anti-inflammatory effects of dexmedetomidine on positive outcomes in patients undergoing surgery with spinal caries, attenuating the release of pro-inflammatory biomarkers. Similarly other group.^23^ studied combined the effects of Dexmedetomidine and Propofol and confirmed the improved quality of analgesia, anesthesia, and decrease in the inflammatory cytokine’s expression.

To the best of our knowledge, present study was unique of its kind as it was designed to evaluate the novel use of IV DEX as premedication for reduction of surgical stress leading to decrease intraoperative and postoperative pain and inflammation in patients undergoing tonsillectomies and comparing with patients in control group who received standard regimen by estimating the stress biomarkers quantitatively.

### Limitations:

We investigated 100 patients for inflammatory markers. Further studies should be carried out as Dexmedetomidine is being used widely in multiple surgeries and in ICU.

## CONCLUSION

Dexmedetomidine when administered pre-and intraoperatively in patients undergoing ENT surgeries especially tonsillectomies, moderated inflammatory and stress responses are linked to suppression of inflammatory cytokines.

### Recommendations:

The drug is already being used in many countries for its beneficial effects and should be used more frequently by anesthesia care providers to prevent common problems in the peri-operative period. It is very likely that this drug will come up to take a much more prominent role in future anesthesia practice.

### Authors Contribution

**KA:** Corresponding author, conceived the idea, drafted, designed, and did statistical analysis & manuscript writing, and is responsible for research integrity.

**AW:** Supervised the study and final approval of the manuscript.

**RF:** Participated to perform the actual study in OT, helped to collect the samples and reviewed the design of study

**QM:** Provided technological support to perform the required procedures, reviewed and proofread the manuscript.
